# A Potential Mechanism Underlying the Therapeutic Effects of Progesterone and Allopregnanolone on Ketamine-Induced Cognitive Deficits

**DOI:** 10.3389/fphar.2021.612083

**Published:** 2021-03-08

**Authors:** Ting Cao, MiMi Tang, Pei Jiang, BiKui Zhang, XiangXin Wu, Qian Chen, CuiRong Zeng, NaNa Li, ShuangYang Zhang, HuaLin Cai

**Affiliations:** ^1^ Department of Pharmacy, Second Xiangya Hospital, Central South University, Changsha, China; ^2^ Institute of Clinical Pharmacy, Second Xiangya Hospital, Central South University, Changsha, China; ^3^ Department of Pharmacy, Xiangya Hospital of Central South University, Changsha, China; ^4^ Institute of Hospital Pharmacy, Xiangya Hospital, Central South University, Changsha, China; ^5^ Institute of Clinical Pharmacology, Jining First People’s Hospital, Jining Medical University, Jining, China

**Keywords:** cognitive deficits, neuroprotection, PGRMC1 signaling, progesterone, allopregnanolone, ketamine

## Abstract

Ketamine exposure can model cognitive deficits associated with schizophrenia. Progesterone (PROG) and its active metabolite allopregnanolone (ALLO) have neuroprotective effects and the pathway involving progesterone receptor membrane component 1 (PGRMC1), epidermal growth factor receptor (EGFR), glucagon-like peptide-1 receptor (GLP-1R), phosphatidylinositol 3 kinase (PI3K), and protein kinase B (Akt) appears to play a key role in their neuroprotection. The present study aimed to investigate the effects of PROG (8,16 mg kg^−1^) and ALLO (8,16 mg kg^−1^) on the reversal of cognitive deficits induced by ketamine (30 mg kg^−1^) via the PGRMC1 pathway in rat brains, including hippocampus and prefrontal cortex (PFC). Cognitive performance was evaluated by Morris water maze (MWM) test. Western blot and real-time quantitative polymerase chain reaction were utilized to assess the expression changes of protein and mRNA. Additionally, concentrations of PROG and ALLO in plasma, hippocampus and PFC were measured by a liquid chromatography-tandem mass spectrometry method. We demonstrated that PROG or ALLO could reverse the impaired spatial learning and memory abilities induced by ketamine, accompanied with the upregulation of PGRMC1/EGFR/GLP-1R/PI3K/Akt pathway. Additionally, the coadministration of AG205 abolished their neuroprotective effects and induced cognitive deficits similar with ketamine. More importantly, PROG concentrations were markedly elevated in PROG-treated groups in hippocampus, PFC and plasma, so as for ALLO concentrations in ALLO-treated groups. Interestingly, ALLO (16 mg kg^−1^) significantly increased the levels of PROG. These findings suggest that PROG can exert its neuroprotective effects via activating the PGRMC1/EGFR/GLP-1R/PI3K/Akt pathway in the brain, whereas ALLO also restores cognitive deficits partially via increasing the level of PROG in the brain to activate the PGRMC1 pathway.

## Introduction

Cognitive deficits have been recognized as a core feature of first-episode and drug-naive schizophrenia patients (Aas et al., 2014; Chu et al., 2019). Evidence indicates that cognitive symptoms in schizophrenia involve alterations of the function of hippocampus (Driesen et al., 2008) and prefrontal cortex (PFC) (Blot et al., 2015), particularly during working memory tasks (Song et al., 2018). Cognitive deficits can strongly predict long-term functional disability in schizophrenia patients, but current antipsychotic treatments lack efficacy for improving cognition in patients (Hill et al., 2010). New adjunctive procognitive drugs are urgently needed and are pivotal for achieving robust cognitive and functional improvement in schizophrenia. Evidence supports that antagonists of NMDA receptors such as PCP, ketamine or MK801 can produce cognitive deficits manifested as relevant to schizophrenia along with certain pathological disturbances seen in the illness (Neill et al., 2010).

Increasing evidence suggests that neurosteroids have neuroprotective properties on the central nervous system (Rajagopal et al., 2018). Previous studies have shown that lack of steroid hormones has an important role in the development of neurological diseases including schizophrenia (Moore et al., 2013), Parkinson’s disease (Nezhadi et al., 2016). It has been demonstrated that the neurosteroids progesterone (PROG) and allopregnanolone (ALLO) exert several functional effects in the brain, such as neuroprotection against some nervous system diseases, including traumatic brain injury (TBI) (Si et al., 2013) and spinal cord injury (Cooke et al., 2013) and schizophrenia-related cognitive dysfunction (Cai et al., 2018a). The underlying mechanisms and the targets of their neuroprotective effects have not been elucidated. As the major active metabolite of PROG, ALLO has been shown to have neuroprotective properties both *in vitro* (Frank and Sagratella, 2000) and *in vivo* (Morali et al., 2011). The prevailing view holds that PROG exerts its neuroprotective effects through multiple receptors: classical progesterone receptors (Pgr), PGRMC1, membrane progesterone receptors (mPR), and GABA_A_ receptors after conversion to ALLO (Cooke et al., 2013; Guennoun et al., 2015).

Progesterone receptor membrane component 1 (PGRMC1), also called 25-Dx, is a multiprotein complex highly expressed in the brain, especially in the hippocampus (Rohe et al., 2009). One of the appealing features of PGRMC1 is its high affinity for PROG and other steroids, which can promote cell survival and damage resistance (Losel et al., 2008). Accumulating evidence supports that PGRMC1 has unique effects in mediating the effects of PROG in preventing apoptosis and promoting cell proliferation and survival (Liu et al., 2009; Peluso et al., 2009). Specifically, it has been demonstrated that increased proliferation induced by PROG in neuroprogenitor cells from the adult rat hippocampus is mediated through PGRMC1 since these cells lack Pgr and that proliferation is inhibited after treatment with PGRMC1 siRNA (Liu et al., 2009). Likewise, treatment with PROG after spinal cord injury can upregulate PGRMC1 without affecting Pgr expression, and this neuroprotective role of PROG through PGRMC1 can also occur in the brain following TBI (Guennoun et al., 2008).

The PI3K/Akt signaling pathway is known to be pivotal for cell survival and the maintenance of several neuronal functions, such as memory formation and potentiation (Zhou et al., 2014). Under certain conditions, the PI3K/Akt pathway can be activated to exert its neuroprotective function by phosphorylating a battery of protein substrates, including Nuclear factor erythroid-2-related factor 2 (Nrf2), caspase-3/9, cAMP response element-binding protein (CREB) and brain-derived neurotrophic factor (BDNF). It is notable that PGRMC1 is able to activate intracellular Akt signaling in cancer (Hand and Craven, 2003) through the epidermal growth factor receptor (EGFR) tyrosine kinase (Aizen and Thomas, 2015), the typical trafficking target for PGRMC1. Moreover, increased PGRMC1-to-Akt activation could increase survival signaling in ER (Estrogen receptor)-negative tumors (Craven, 2008). A recent study reported that the knockdown of PGRMC1 and AG205 treatment both potentiated insulin-mediated phosphorylation of the IR signaling mediator Akt (Hampton et al., 2018).

Cogent evidence has revealed that the PI3K/Akt pathway is a putative downstream signaling pathway regulated by EGFR (MacDonald et al., 2003) and GLP-1R to elicit multiple biological responses, especially cognitive function (Zhu et al., 2016; Xie et al., 2018). Intriguingly, PGRMC1 co-precipitates and co-localizes with EGFR in cytoplasmic vesicles in cells (Ahmed et al., 2010) and also serves as a novel component of the liganded GLP-1R complex (Zhang et al., 2014). Therefore, it was likely that PGRMC1 dually regulates the PI3K/Akt signaling pathway by combining with GLP-1R and EGFR.

Taken together, the present study aimed to figure out 1) whether the PGRMC1/EGFR/GLP-1R/PI3K/Akt pathway underlies the mechanism of the neuroprotective effect of PROG against ketamine-induced cognitive dysfunction and 2) how ALLO exerts its neuroprotective function in the ketamine-induced model. The mechanisms of the potential effects were validated via AG205, a specific inhibitor of PGRMC1.

## Materials and Methods

### Animals

To avoid possible influence of cyclic, systemic PROG fluctuation caused by estrous cycle (Grassi et al., 2011; Di Mauro et al., 2015), only male Sprague–Dawley rats were used in our study. Rats weighting between 150 and 200 g (approximately 5 weeks old) were purchased from Hunan Slack Jingda Experimental Animal Co., Ltd. (Changsha, Hunan). In experiment 1 and 2, 18 rats (*n* = 3/group) were used to assess the effect of PROG and ALLO on PGRMC1 expression in basal conditions. In experiment 3, 12 rats (*n* = 6/group) were used for the validation of the inhibitory effects of AG205 on PGRMC1. In experiment 4, a total of 49 rats were used for exploring the potential mechanism underneath the therapeutic effects of PROG and ALLO against ketamine-induced cognitive deficits.

All rats were housed with free access to food and water, under the conditions of a light-dark cycle (12 h/12 h), humidity at 45–50%, and room temperature (24–25°C). The animal housing conditions were set as follows: home cage size at 470 mm × 312 mm × 260 mm (length×width×height), three rats per cage, poplar sawdust bedding. The water bottle was fulfilled with purified water daily. The beddings were changed and cages were cleaned and disinfected every 2 days. All rats were acclimatized for 1 week before experimentation. The animal research protocol was approved by the local Ethics Committee of the Second Xiangya Hospital of Central South University (Approval No. 2020008). All efforts were made to reduce animal suffering and the number of animals used.

### Chemicals and Reagents

2-Hydroxypropyl β–cyclodextrin was purchased from Sigma-Aldrich Inc. (St. Louis, MO, United States). HPLC grade acetonitrile (ACN), methanol and methyl tert-butyl ether (MTBE) were supplied by Merck KGaA (Darmstadt, Germany), and 2-propanol (IPA) was provided by Anaqua Chemical Supply Inc. (Wilmington, DE, United States). PROG and ALLO standards were purchased from Sigma-Aldrich (Shanghai, China) and Steraloids Inc. (Wilton, NH, United States), respectively. AG205 (purity ≥ 97.5%) was synthesized by Jining Drug Research and Development Center. PROG (purity ≥ 99.5%) and ALLO (purity ≥ 99.0%) were obtained from Wuhan Chemduro Pharm Co., Ltd. Injectable ketamine was acquired from the Second Xiangya Hospital of Central South University. Based on most preclinical studies (Kumon et al., 2000; Wali et al., 2014; Andrabi et al., 2017), PROG or ALLO at 8 mg kg^−1^ or 16 mg kg^−1^ was mostly adopted and further proved to exert neuroprotective effects in rats with brain injury. The dose used for the PROG and ALLO treatments was based on previous results suggesting that 8 and 16 mg kg^−1^ of PROG and ALLO were optimal for facilitating recovery of cognitive outcome in CNS impairment (Goss et al., 2003; Djebaili et al., 2004; Morali et al., 2011). Moreover, we chose five-day regime with one injection per day based on the two considerations: 1) good cognitive and sensory recovery could be obtained when 5 days of post-injury neurosteroid injections are provided (Goss et al., 2003; Djebaili et al., 2004) and 2) five-day administration was employed in building the animal model of ketamine-induced cognitive deficits and the treatment with neurosteroids should be conducted accordingly.

Enzyme activity assay kits for superoxide dismutase (SOD), catalase (CAT) and glutathione peroxidase (GSH-Px) were purchased from Nanjing Jiancheng Bioengineering Institute. The Pentobarbital sodium solutions used for surgical procedures were purchased as commercial preparations for veterinary use.

### Preparation of Drug Solution

Five ampoules of ketamine injection (0.1 g/2 ml) were diluted with an additional volume of 100 ml of 0.9% saline water to achieve a final concentration of 4.5 mg ml^−1^. PROG (8 mg ml^−1^), ALLO (8 mg ml^−1^) and AG205 (7.3 mg ml^−1^) were initially dissolved in 5% (v/v) ethanol and then further diluted in 0.9% saline water containing 22.5% 2-hydroxypropyl β–cyclodextrin to obtain the final concentration. All the solutions were injected intraperitoneally. Due to lack of evidence in application of AG205 in animal models, we calculated the AG205 dose converted from PROG according to their molecular docking score binding to PGRMC1 (Detailed information of the calculation process is illustrated in Supplementary Tables S1–S3).

### Experimental Schedule

**FIGURE 1:**
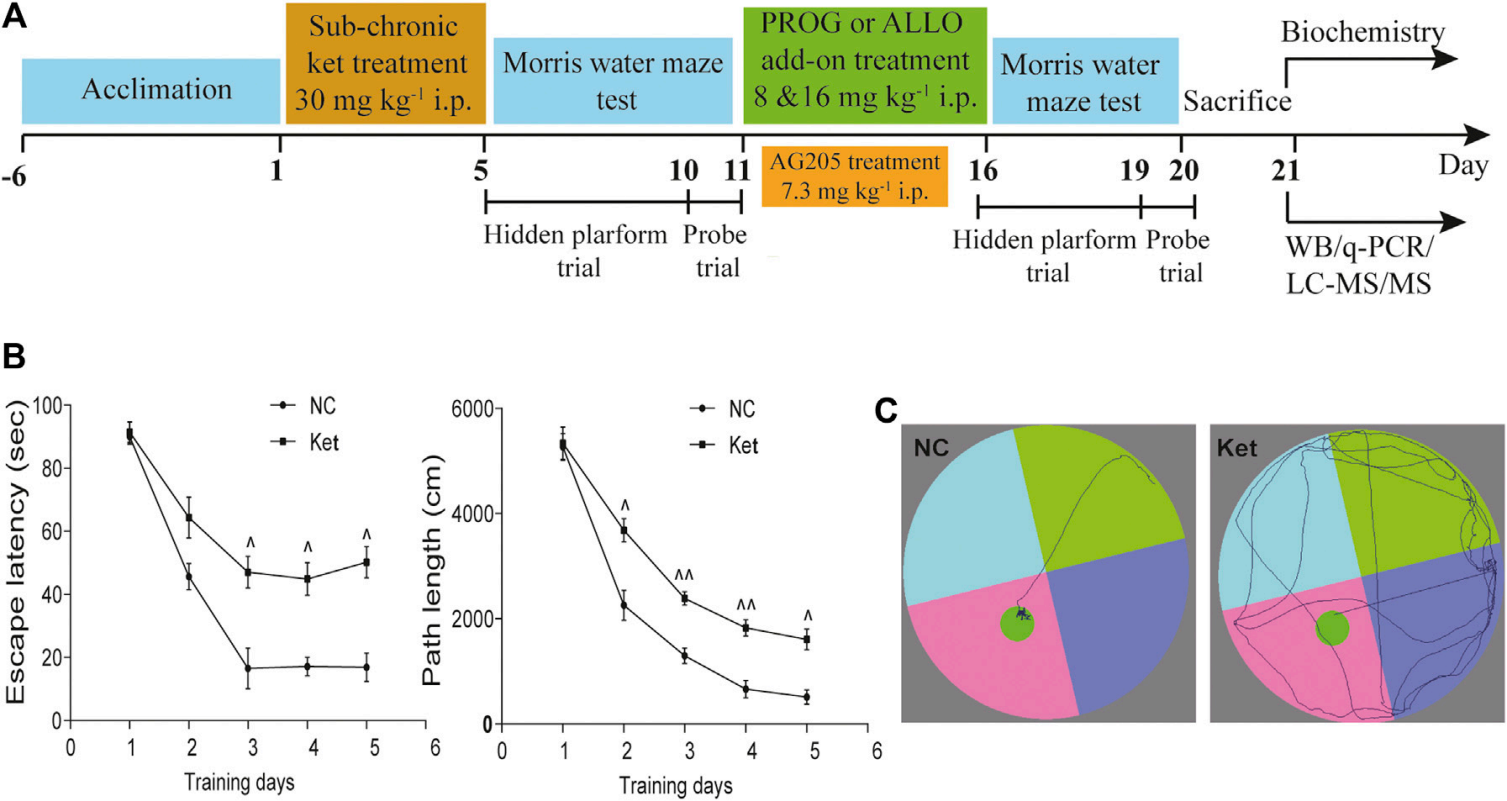
Cognitive performance in the hidden platform trials of MWM test after ketamine exposure. **(A)** A diagram of the time course illustrating when the procedures took place. **(B)** Learning ability manifested as escape latency and path length of ketamine-exposed rats (*n* = 42) vs. NC (*n* = 7). **(C)** The swimming traces of the rats are illustrated (NC vs. Ket). ^^^ *p* < 0.05, ^^^^ *p* < 0.01, and ^^^^^ *p* < 0.001 for Ket vs. NC.

In experiment 1 (Supplementary Figure S1i) and 2 (Supplementary Figure S1ii), rats were randomly assigned to vehicle- and PROG- or ALLO-treated groups. The vehicle-treated animals received 0.9% saline water containing 22.5% 2-hydroxypropyl β–cyclodextrin and the PROG- or ALLO-treated groups received PROG (8 or 16 mg kg^−1^) or ALLO (8 or 16 mg kg^−1^) daily for five consecutive days. In experiment 3 (Supplementary Figure S1iii), animals were randomly assigned to vehicle and AG205-treated groups. Intraperitoneal injection of AG205 (7.3 mg kg^−1^) was conducted daily for five consecutive days. Before sacrificing, a five-day MWM task was carried out to evaluate the learning ability and spatial memory of rats, including a four-day hidden platform trial and probe trial on the fifth day. In experiment 4 (Figure 1A), the whole cohort was divided in two main groups, referred to normal control (NC) group (*n* = 7) and ketamine-exposed rats (Ket, *n* = 42). Firstly, in order to mimic schizophrenia-like cognitive deficits in rats, ketamine was given intraperitoneally daily at a dose of 30 mg kg^−1^ for five consecutive days (Day 1–Day 5). Subsequently, MWM task (Day 5–Day 11) was utilized to test spatial memory and learning ability to further evaluated the effects of ketamine on cognitive function. Ketamine-exposed rats were randomly assigned to six groups (*n* = 7, each group) with different treatments: 1) vehicle; 2) PROG (8 mg kg^−1^); 3) PROG (16 mg kg^−1^); 4) ALLO (8 mg kg^−1^); 5) ALLO (16 mg kg^−1^); and 6) PROG (8 mg kg^−1^)+AG205. They were administered intraperitoneal injection of vehicle (0.9% saline containing 22.5% 2-hydroxypropyl β–cyclodextrin), PROG (8 mg kg^−1^), PROG (16 mg kg^−1^), ALLO (8 mg kg^−1^ per day), ALLO (16 mg kg^−1^), PROG (8 mg kg^−1^)+AG205 (7.3 mg kg^−1^) daily for five consecutive days (Day 11–Day 16). Before sacrificing, the MWM task were carried out from day 16 to day 20. All rats were fasted for 12 h before sacrifice. The rats were anesthetized with 2% pentobarbital sodium solution (0.2 ml/100 g), and tissue samples from the PFC and hippocampus were collected and frozen immediately in liquid nitrogen. Blood was collected from the truncal vessel in EDTA anticoagulation vacuum tubes. The plasma was centrifuged at 4°C, 3,000 rpm for 15 min stored at −80°C before analysis.

### Morris Water Maze

In order to evaluate the cognitive performance including learning and spatial memory, we performed the MWM task experiments. The water maze (Gene&I instruments, Beijing, China, model number: CSI-MZ-WM-H) consisted of a circular pool, 1.8 m in diameter, 60 cm in height, and 4.7 mm in thickness surrounded by curtains. There were four different geometric shapes positioned on the four walls as spatial cues. The pool was filled with tap water at a depth of approximately 50 cm, and the water temperature was maintained at 24–25°C. Meanwhile, a sufficient amount of edible black pigment was added to obscure the water. An escape platform was fixed in the center of the target quadrant 1.0 cm underneath the surface of the water. Each rat was released along the wall into the center of one of three randomly chosen quadrants of the maze. Trials were recorded and captured using a video tracking system (Topscan system 3.0, CleverSys Inc.) connected to a computer.

As described in previous studies (Moosavi et al., 2007; Moosavi et al., 2012; Wang et al., 2014), in the hidden platform trial, the rats were allowed to swim in the maze for no more than 90 s until it located the hidden platform. If a rat did not reach the platform within 90 s, the experimenter would guide it to the platform, where it was kept for 20 s. There was a 30-s interval before the next trial. A total of three training trials were carried out in one day. In the probe trial phase, the platform was removed from the pool. Each rat was placed in the center of the farthest quadrant from the target quadrant, and each rat performed only one trial. The tracking system recorded the swimming path of each rat within 120 s. After the training trials were finished, the rats were dried with towels before being returned to their cages.

### Western Blot

Proteins were extracted from the collected hippocampus and PFC tissues and concentrations were measured as described previously (Cai et al., 2015). Approximately 20 μg of protein was loaded onto a 10% or 12% sodium dodecyl sulfate-polyacrylamide gel, transblotted onto PVDF membranes, blocked with 5% nonfat milk in TBST (0.1 M Tris–HCl, pH 8.5, 1.5 M NaCl, 0.5% Tween-20) or 5% BSA at room temperature for an hour and incubated overnight at 4°C with a primary antibody diluted to the appropriate concentration. Primary antibodies, including rabbit anti-PGRMC1 polyclonal antibody (12990-1-AP; 1:1,000), rabbit anti-EGFR polyclonal antibody (18986-1-AP; 1:1,000), rabbit anti-GLP-1R polyclonal antibody (26196-1-AP; 1:1,000), mouse anti-PI3K monoclonal antibody (60225-1-lg; 1:5,000), rabbit anti-Akt polyclonal antibody (10176-2-AP; 1:2,000), rabbit anti-CREB polyclonal antibody (12208-1-AP; 1:1,000), mouse anti-Caspase 9 monoclonal antibody (66169-1-Ig; 1:500) were purchased from Proteintech Group (Wuhan, China). Mouse anti-p-EGFR monoclonal antibody (#2236; 1:1,000), rabbit anti-p-PI3K monoclonal antibody (#4228S; 1:1,000), rabbit anti-p-Akt monoclonal antibody (#4060; 1:1,000) and rabbit anti-Caspase 3 monoclonal antibody (#9662; 1:1,000) were purchased from Cell Signaling Technology, Inc. (Boston, United States). Rabbit anti-BDNF monoclonal antibody (ab108319; 1:1,000) was ordered from Abcam (United Kingdom). After washing with TBST, the membranes were incubated with horseradish peroxidase–conjugated goat anti-rabbit IgG (BA1054; 1:5,000; Boster, Wuhan, China) or goat anti-mouse IgG (BA1050; 1:5,000; Boster, Wuhan, China) for 1 h at room temperature. The film signal was digitally scanned and then quantified using ImageJ software (National Institutes of Health, Bethesda, MD, United States). The ratio to β-actin was calculated, and the mean value of the NC group was set at 1.

### Real-Time qPCR and Biochemical Assays

Total RNA from the hippocampus and PFC was isolated with Trizol reagent and then converted to cDNA via the cDNA Synthesis Kit (Life Technologies) according to the manufacturer’s protocol. After the quantification of mRNAs, amplification was performed with the following gene-specific primers: Nfr2, forward: 5′-AGT​GCA​AGG​CGG​AGG​TGA-3′ and reverse: 5′-AGC​CCG​TTG​GTG​AAC​ATA​G-3’; β-actin, forward: 5′- CAT​CCT​GCG​TCT​GGA​CCT​GG-3′ and reverse: 5′-TAA​TGT​CAC​GCA​CGA​TTT​CC-3’. The amplification reaction consisted of an initial activation at 95°C for 15 min and 40 cycles of denaturation at 95°C followed by a 30-s extension at 60°C. The results are presented as the ratio to β-actin and were normalized to the NC group.

The activity of antioxidant enzymes (CAT, SOD, GSH-Px) was measured using commercial enzyme activity assay kits according to the attached protocols.

### Determination of Concentrations of PROG and ALLO

Aliquots of 0.1 g of PFC/hippocampal tissue were homogenized with 1 ml of prechilled methanol using a Bioprep-24 Homogenizer System (speed: 3,500 rpm, time: 30 s, cycles: 3, interval: 30 s). Then, an aliquot of 300 μl of the tissue homogenate or plasma was transferred and mixed with 1,500 μl of methyl tert-butyl ether/methanol (1:1, v/v) extraction solvent, vortexed for 3 min and centrifuged at 20627 *g* for 10 min at 4°C. A volume of 1,500 μl of the resulting supernatant was evaporated to dryness using a centrifugal vacuum concentrator at 4°C. The residue was further resuspended with 100 μl of 2-propanol/acetonitrile/water (21:9:70, v/v/v) solvent mixture. Finally, the concentrations of PROG and ALLO in the tissues and plasma were determined by an LC–MS/MS method as reported elsewhere (Cai et al., 2019). Both lower limit of quantifications (LLOQs) of PROG and ALLO were 0.05 ng/ml for plasma, and were 0.15 ng/g for brain tissue, respectively. For intra-assay, the coefficient of variance (CV) in PROG ranged from 2.4 to 9.6%, whereas CV in ALLO ranged from 3.9 to 5.7%. For inter-assay, the CV in PROG varied between 3.9 and 7.1%, whereas CV in ALLO varied between 4.5 and 8.3%.

### Statistical Analysis

The data are presented as the mean ± SD and were analyzed using GraphPad Prism 8.0 (GraphPad Software, San Diego, CA, United States). After ketamine exposure, performance in the hidden platform trials in the MWM was analyzed using repeated measures analysis of variance (RM-ANOVA), with treatment and time as two independent variables, followed by Dunnett’s *t*-test for post hoc test. For parameters in the first probe trial, Mann-Whitney *U* test was used to evaluate ketamine induced behavioral changes when building the animal model of cognitive deficits. Differences in the behavioral tests, mRNA expression, protein expression and the enzyme activities in hippocampus across groups were determined using Kruskal-Wallis one-way analysis of variance followed by post hoc Dunn’s multiple comparisons test. Statistical significance was considered at *p* < 0.05.

## Results

### The Effects of PROG, ALLO and AG205 on PGRMC1 Expression in Basal Conditions

As illustrated in Supplementary Figure S1, compared with NC group, sub-chronic PROG (16 mg kg^−1^) treatment markedly downregulated the PGRMC1 expression both in hippocampus (*H* = 5.956, *p* = 0.0250; post hoc *p* = 0.0341) and PFC (*H* = 6.252, *p* = 0.0143; post hoc *p* = 0.0270). However, the inhibitory effects of PROG (8 mg kg^−1^) and ALLO on PGRMC1 did not approach significance (all *p* > 0.05). As depicted in Supplementary Figure S1D, compared with NC group, AG205-treated rats exhibited significantly longer escape latency on the 2nd, third, fourth and fifth day respectively. For escape latency, an effect of treatment [*F* (1, 25) = 92.76, *p* < 0.0001] and an effect of time [*F* (4, 25) = 297.4, *p* < 0.0001], but no interaction [*F* (4, 25) = 0.9234, *p* = 0.2632]. while in the probe trial, AG205 treatment reduced the number of target crossings (*U* = 0, *p* = 0.0022), the permanence time (PT) (*U* = 0, *p* = 0.0022) and PT% in the target quadrant [*U* = 0, *p* = 0.0022]. Western blot experiment was utilized to measure the level of PGRMC1 in rat brain including hippocampus and PFC. As shown in Supplementary Figure S1C, AG205 markedly downregulated the expression of PGRMC1 in hippocampus (*U* = 0, *p* = 0.0022) and PFC (*U* = 0, *p* = 0.0022) compared with normal rats, which provided support for the role of AG205 in co-administration with PROG.

### Ketamine Exposure Impaired Memory Performance in the MWM Test

First, we explored whether rats exposed to 30 mg kg^−1^ ketamine sub-chronically for 5 days show memory impairment in the MWM test. In the hidden platform phase, compared with NC group, ketamine-exposed rats exhibited significantly prolonged escape latency and path lengths on the third, fourth and fifth day respectively (Figure 1B). For escape latency, an effect of treatment [*F* (1, 250) = 14.40, *p* = 0.0002] and an effect of time [*F* (4, 250) = 15.22, *p* < 0.0001] were observed without interaction [*F* (4, 250) = 0.9594, *p* = 0.4304]. Similarly, for path lengths there were an effect of treatment [*F* (1, 770) = 16.00, *p* < 0.0001] and an effect of time [*F* (4, 770) = 41.40, *p* < 0.0001], but no interaction [*F* (4, 770) = 0.9234, *p* = 0.4497] existed. However, in the probe trial, the ketamine-exposed rats showed significantly less target quadrant crosses (*U* = 9.00, *p* < 0.0001, Cohen’s *d* = 1.363) and PT in the target area (*U* = 61.50, *p* = 0.0080, Cohen’s *d* = 0.745) and a reduced PT% in the target area (*U* = 67.50, *p* = 0.0136, Cohen’s *d* = 0.686) (Table 1).

**TABLE 1:** Parameters of the probe trial in MWM test (mean ± SD).

Experiments	Groups	Parameters		
		Number of the target crossings^a^	PT in the target quadrant (sec)^b^	Percentage (%) of PT in the target quadrant^c^
^A^Ketamine exposure	NC (*n* = 7)	8.57 ± 3.11	24.21 ± 5.02	35.68 ± 11.23
	Ket (*n* = 42)	2.22 ± 1.55^^^^^	16.83 ± 6.50^^^^	24.85 ± 8.43^^^
^B^Add-on treatments (n = 7/group)	NC	5.14 ± 1.57	41.70 ± 5.09	35.74 ± 6.73
	Ket	1.71 ± 0.95^^^^	22.46 ± 7.47^^^	21.02 ± 3.81^^^^
	PROG (8 mg kg^−1^)	4.43 ± 0.79^**^	36.51 ± 8.57*	35.24 ± 4.77*
	PROG (16 mg kg^−1^)	2.29 ± 0.95	26.70 ± 6.07	27.78 ± 4.17
	ALLO (8 mg kg^−1^)	4.00 ± 0.82^*^	37.13 ± 8.71*	39.79 ± 8.59**
	ALLO (16 mg kg^−1^)	3.71 ± 1.11^*^	33.17 ± 9.71*	34.00 ± 9.41*
	PROG (8 mg kg^1^) +AG205	2.57 ± 0.97^#^	22.63 ± 8.38^#^	17.71 ± 8.43^##^

**A** Before add-on treatments, ketamine strongly decreased the number of target crossings [*U* = 9.00, *p* < 0.0001, Cohen’s *d* = 1.363] along with permanence time [*U* = 61.50, *p* = 0.0080, Cohen’s *d* = 0.745] in the target area and reduced percentage of permanence time in target area [*U* = 67.50, *p* = 0.0136, Cohen’s *d* = 0.686] in rats, as revealed by Mann-Whitney *U* test.

**B** The effects of add-on treatments on MWM performance. Kruskal-Wallis test was used for analysis followed by Dunn’s multiple comparisons test for post hoc test. Data are expressed as mean ± SD, *n* = 7 for each group. ^^^ *p* < 0.05, ^^^^ *p* < 0.01, and ^^^^^ *p* < 0.0001 for Ket vs. NC. ^*^ *p* < 0.05, ^**^ *p* < 0.01 and ^***^ *p* < 0.0001 for add-on of PROG and ALLO vs. Ket. ^#^ *p* < 0.05, ^##^ *p* < 0.01 and ^###^ *p* < 0.0001 for PROG (8 mg kg^−1^)+AG205 vs. PROG (8 mg kg^−1^).

^a^ Number of the target crossings [*H* = 29.62, *p* < 0.0001, Cohen’s *d* = 2.267].

^b^ PT in the target quadrant [*H* = 22.79, *p* = 0.0009, Cohen’s *d* = 1.632].

^c^ Percentage (%) of PT in the target quadrant [*H* = 28.85, *p* < 0.0001, Cohen’s *d* = 2.185].

In order to avoid the influences of the performance during the first MWM learning in ketamine-treated animals on the results of the second MWM. As shown in Table 2, it indicated that there are no differences in escape latency (*H* = 0.6189, *p* = 0.9871, Cohen’s *d* = 0.744) and path length (*H* = 0.8866, *p* = 0.9712, Cohen’s *d* = 0.718) on the third day among the groups randomly distributed with the animals during first MWM test.

**TABLE 2:** Performance of ketamine-treated rats randomly assigned to each group before add-on treatments (mean ± SD).

^A^ Ketamine exposure	Groups	Parameters on the 3rd day during the first MWM learning trial	
		Escape latency (sec)^a^	Path length (cm)^b^
Ket-exposed rats (*n* = 7/group)	Ket	51.00 ± 13.56	1699.0 ± 283.7
	PROG (8 mg kg^−1^)	49.14 ± 17.68	1574.0 ± 361.6
	PROG (16 mg kg^−1^)	50.00 ± 24.15	1714.0 ± 347.6
	ALLO (8 mg kg^−1^)	50.14 ± 26.44	1570.0 ± 331.1
	ALLO (16 mg kg^−1^)	51.43 ± 22.43	1652.0 ± 398.3
	PROG (8 mg kg^−1^)+AG205	49.86 ± 16.89	1646.0 ± 394.1

Kruskal-Wallis test was used for analysis followed by Dunn's multiple comparisons test for post hoc test. Data are expressed as mean ± SD, *n* = 7 for each experimental group.

^a^ There are no differences in escape latency [*H* = 0.6189, *p* = 0.9871, Cohen’s *d* = 0.744].

^b^ There are no differences in path length [*H* = 0.8866, *p* = 0.9712, Cohen’s *d* = 0.718] on the third day among the ketamine-treated groups during first MWM test before add-on treatments.

### Neuroprotective Effects of PROG and ALLO on MWM Performance

**FIGURE 2:**
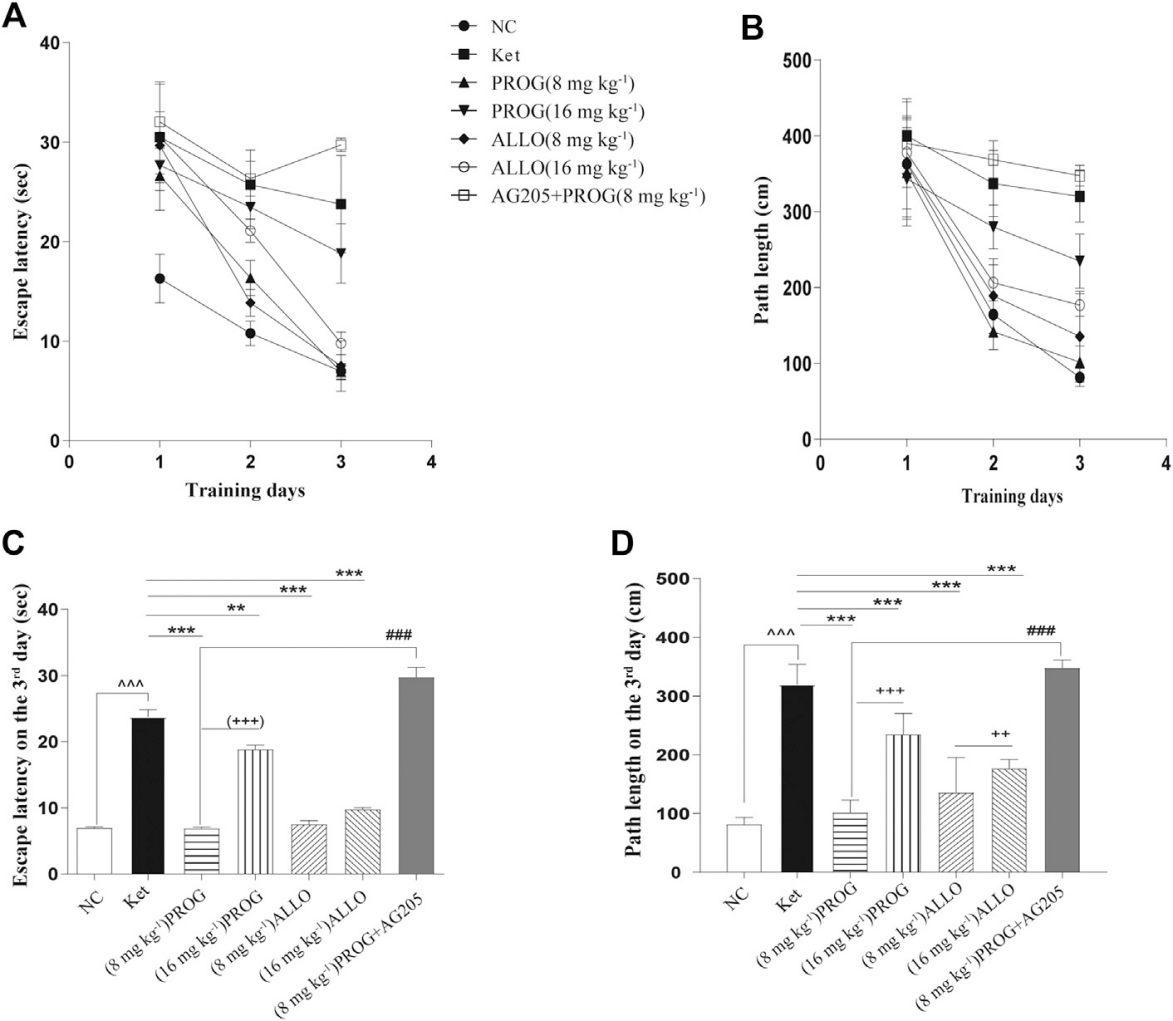
Cognitive performance in the hidden platform trials of MWM test after add-on PROG and ALLO treatments. **(A)** Escape latency **(B)** Path length **(C)** Escape latency on the third day and **(D)** path lengths on the third day were shortened by PROG (all *p* < 0.01) or ALLO treatment (all *p* < 0.0001) in comparison with ketamine treatment. Data are expressed as mean ± SD, *n* = 7 for each experimental group. ^*^ *p* < 0.05, ^**^ *p* < 0.01 and ^***^ *p* < 0.001 for add-on of PROG and ALLO vs. Ket. ^#^ *p* < 0.05, ^##^ *p* < 0.01 and ^###^ *p* < 0.001 for PROG (8 mg kg^−1^)+AG205 vs. PROG (8 mg kg^−1^). ^+^ *p* < 0.05, ^++^ *p* < 0.01 and ^+++^ *p* < 0.001 for PROG (16 mg kg^−1^) vs. PROG (8 mg kg^−1^) and ALLO (16 mg kg^−1^) vs. ALLO (8 mg kg^−1^).

Cognitive performance was evaluated by MWM task after add-on PROG and ALLO treatments. In the hidden platform trial, path length and escape latency were robustly shortened after PROG and ALLO treatments (Figures 2A,B). For path length, an effect of treatment [*F* (6, 420) = 169.7, *p* < 0.0001], an effect of time [*F* (2, 420) = 664.3, *p* < 0.0001], and an interaction between factors [*F* (12, 420) = 30.07, *p* < 0.0001]. For escape latency, an effect of treatment [*F* (6, 420) = 343.2, *p* = 0.0002], an effect of time [*F* (2, 420) = 857.7, *p* < 0.0001] and an interaction between factors [*F* (12, 420) = 50.30, *p* < 0.0001]. Not all parameters related to cognitive function were significantly affected in a dose-dependent manner. In the probe trial, as compared with ketamine group, treatments with PROG (8 mg kg^−1^) or ALLO (8/16 mg kg^−1^) led to a significant increase in the number of target crossings (*H* = 29.62, *p* < 0.0001, Cohen’s *d* = 2.267), less PT in the target area (*H* = 22.79, *p* = 0.0009, Cohen’s *d* = 1.632) and a reduced PT% in the target area (*H* = 28.85, *p* < 0.0001, Cohen’s *d* = 2.185) (Table 1). In order to reflect the variances between ketamine and NC groups more directly, Figures 2C,D indicated that escape latency on the third day (*H* = 42.49, *p* < 0.0001, Cohen’s *d* = 5.147) and path lengths on the third day (*H* = 45.40, *p* < 0.0001, Cohen’s *d* = 7.786) were shortened by treatments of PROG or ALLO in comparison with ketamine treatment.

### Suppression of the PGRMC1 Signaling Pathway by Ketamine and the Reversal Effects of PROG and ALLO

To explore the potential modulatory effects of ketamine, PROG and ALLO add-on treatments on the PGRMC1/EGFR/GLP-1R/PI3K/Akt signaling pathway, the protein expression of the five key factors (PGRMC1, EGFR, GLP-1R, PI3K, Akt) and their phosphorylated forms (p-EGFR, p-PI3K, p-Akt) in the hippocampus and PFC was compared among groups.

**FIGURE 3:**
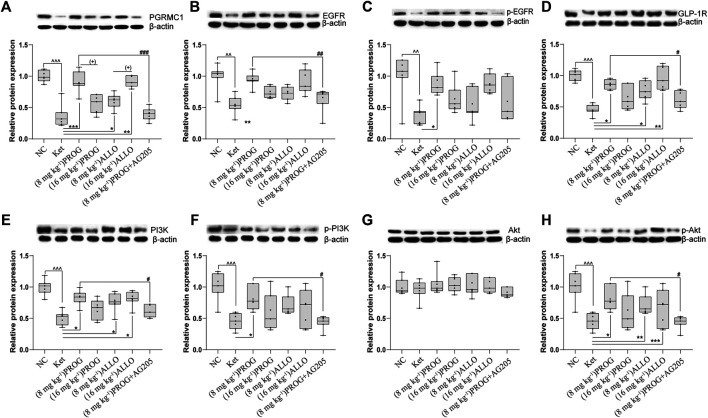
Basal protein expression profile of the PGRMC1/EGFR/GLP-1R/PI3K/Akt pathway in the hippocampus. **(A)** PGRMC1, **(B)** EGFR, **(C)** *p*-EGFR, **(D)** GLP-1R, **(E)** PI3K, **(F)** p-PI3K, **(G)** Akt, **(H)** p-Akt. Data are expressed as mean ± SD, *n* = 7 for each experimental group. ^^^ *p* < 0.05, ^^^^ *p* < 0.01, and ^^^^^ *p* < 0.001 for Ket vs. NC. ^*^ *p* < 0.05, ^**^ *p* < 0.01 and ^***^ *p* < 0.001 for add-on of PROG and ALLO vs. Ket. ^#^ *p* < 0.05, ^##^ *p* < 0.01 and ^###^ *p* < 0.001 for PROG (8 mg kg^−1^)+AG205 vs. PROG (8 mg kg^−1^). ^+^ *p* < 0.05, ^++^ *p* < 0.01 and ^+++^ *p* < 0.001 for PROG (16 mg kg^−1^) vs. PROG (8 mg kg^−1^) and ALLO (16 mg kg^−1^) vs. ALLO (8 mg kg^−1^). The relative expression data are presented as the ratio to the β-actin protein level. **Note:** In our study, the prototype protein and its phosphorylated form were separated in one gel simultaneously. Therefore, the loading control images of β-actin are re-used for illustrative purposes in **B** and **C**, **E** and **F**, **G** and **H**.

In the hippocampus, sub-chronic ketamine treatment significantly downregulated the protein expression of the five key factors (PGRMC1: *H* = 38.01, *p* = 0.0002; post hoc *p* = 0.0001; GLP-1R: *H* = 31.91, *p* = 0.0002; post hoc *p* = 0.0003; EGFR: *H* = 26.80, *p* = 0.0002; post hoc *p* = 0.0014; PI3K: *H* = 30.78, *p* < 0.0001; post hoc *p* = 0.0005; p-EGFR: *H* = 32.44, *p* < 0.0001; post hoc *p* = 0.0014; p-PI3K: *H* = 33.76, *p* < 0.0001, post hoc *p* < 0.0001; p-Akt: *H* = 39.09, *p* < 0.0001; post hoc *p* < 0.0001) without affecting Akt (*p* > 0.9999) compared to NC group (Figure 3). Intriguingly, PROG administration did not activate the PGRMC1 signaling pathway in a dose-dependent manner. Only low dose of PROG significantly upregulated the protein expression of PGRMC1 pathway (PGRMC1, *p* = 0.0004; GLP-1R, *p* = 0.0214; EGFR, *p* = 0.0046; PI3K, *p* = 0.0298; *p*-EGFR, *p* = 0.0399; p-PI3K, *p* = 0.0159; p-Akt, *p* = 0.0373) without affecting Akt (*p* > 0.9999) compared to Ket group (Figure 3). In contrast, both doses of ALLO administration increased the protein expression of PGRMC1 (*p* = 0.0130, *p* = 0.0030), GLP-1R (*p* = 0.0169, *p* = 0.0039), PI3K (*p* = 0.0329, *p* = 0.0237), p-Akt (*p* = 0.0015, *p* = 0.0005). There was a significant difference in PGRMC1 expression between the low dose and high dose of PROG (PGRMC1, *p* = 0.0144), so as the case for ALLO (PGRMC1, *p* = 0.0176) (Figure 3A). Similar with the hippocampus, the synchronous expression of proteins involved in the PGRMC1 signaling pathway also occurred in the PFC, and the results are shown in Supplementary Figure S2.

### Add-on PROG and ALLO Treatments Alleviated the Modulatory Effects of Ketamine on Downstream Molecules.

**FIGURE 4:**
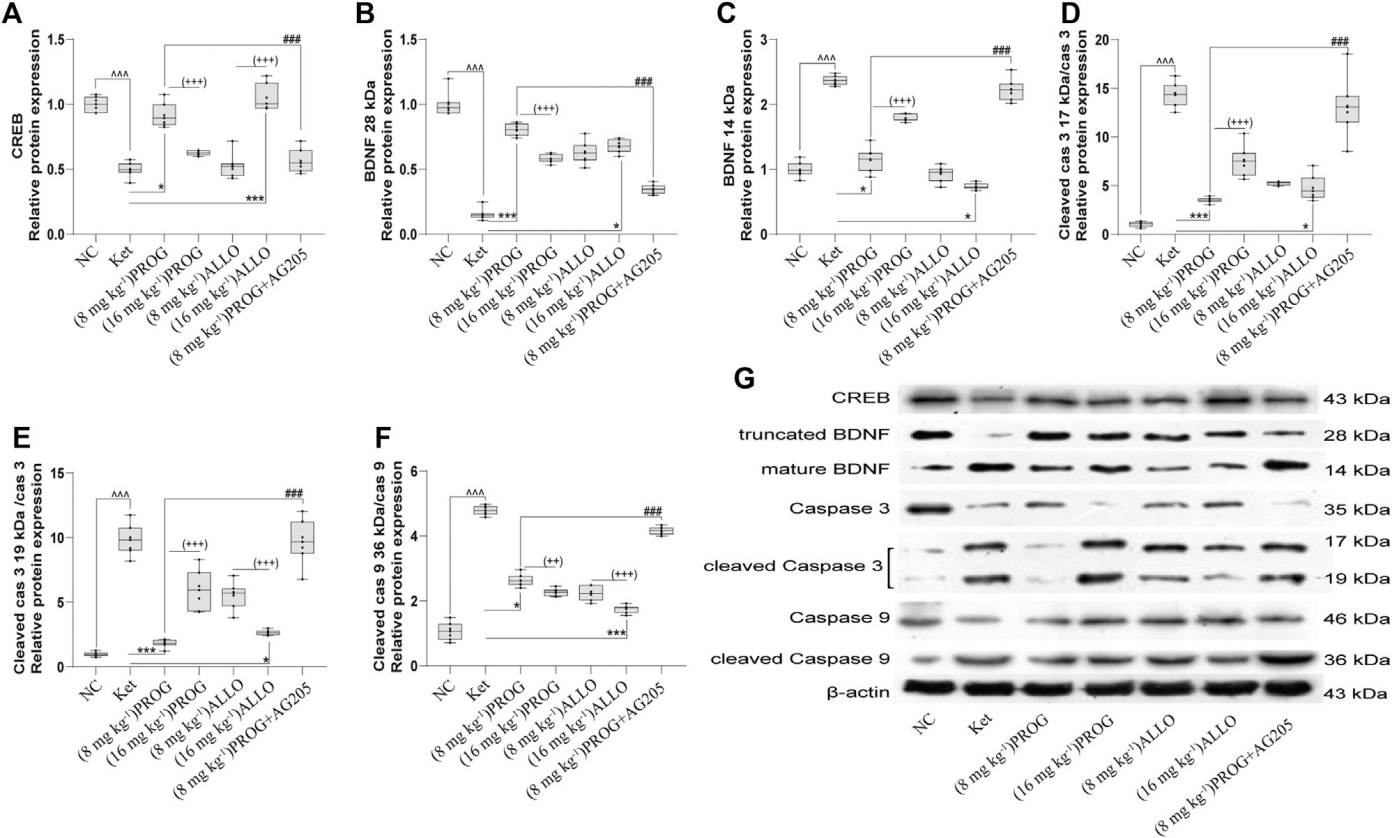
Basal CREB/BDNF/caspase-3/9 protein expression in the hippocampus. **(A)** CREB, **(B)** truncated BDNF, **(C)** mature BDNF, **(D)** cleaved caspase-3 (17 kDa)/caspase-3, **(E)** cleaved caspase-3 (19 kDa)/caspase-3, **(F)** cleaved caspase-9/caspase-9. A representative blot is shown in **(G)**. Data are expressed as mean ± SD, *n* = 7 for each experimental group. ^^^ *p* < 0.05, ^^^^ *p* < 0.01, and ^^^^^ *p* < 0.001 for Ket vs. NC. ^*^ *p* < 0.05, ^**^ *p* < 0.01 and ^***^ *p* < 0.001 for add-on of PROG and ALLO vs. Ket. ^#^ *p* < 0.05, ^##^ *p* < 0.01 and ^###^ *p* < 0.001 for PROG (8 mg kg^−1^)+AG205 vs. PROG (8 mg kg^−1^). ^+^ *p* < 0.05, ^++^ *p* < 0.01 and ^+++^ *p* < 0.001 for PROG (16 mg kg^−1^) vs. PROG (8 mg kg^−1^) and ALLO (16 mg kg^−1^) vs. ALLO (8 mg kg^−1^). The relative expression data are presented as the ratio to the β-actin protein level.

To gain insights into the PGRMC1-regulated signaling pathway, the protein expression of downstream cognitive function-related molecules regulated by the PGRMC1 signaling pathway (including CREB, caspase-3/9, their cleaved forms, and BDNF) were assessed by Western blot experiments in the rat hippocampus and PFC after add-on treatments. As shown in Figures 4A–F, the hippocampal protein expression of CREB (*H* = 39.75, *p* < 0.0001; post hoc *p* = 0.0008) and truncated BDNF (*H* = 44.92, *p* < 0.0001; post hoc *p* < 0.0001) was significantly reduced and the protein levels of mature BDNF (*H* = 42.13, *p* < 0.0001; post hoc *p* = 0.0001) and the ratio of cleaved caspase-3/9 (including cleaved caspase-3 (17 kDa)/caspase-3 [*H* = 44.76, *p* < 0.0001; post hoc *p* < 0.0001], cleaved caspase-3 (19 kDa)/caspase-3 (*H* = 44.80, *p* < 0.0001; post hoc *p* < 0.0001), and cleaved caspase-9/caspase-9 (*H* = 45.91, *p* < 0.0001; post hoc *p* < 0.0001) were upregulated by ketamine exposure.

PROG and ALLO may regulate hippocampal protein expression in a dose-dependent manner but in a contradictory way. Add-on treatment with PROG at 8 mg kg^−1^ or ALLO at 16 mg kg^−1^ markedly mitigated the increased level of mature BDNF (PROG, *p* = 0.0171; ALLO, *p* = 0.0216) and the ratios of cleaved caspase-3 (PROG, *p* = 0.0008; ALLO, *p* = 0.0310) and cleaved caspase-9 (PROG, *p* = 0.0255; ALLO, *p* < 0.0001) induced by ketamine. Meanwhile, PROG (8 mg kg^−1^) or ALLO (16 mg kg^−1^) also ameliorated the decreases in CREB (*p* = 0.0176; *p* = 0.0003) and truncated BDNF (*p* = 0.0001; *p* = 0.0144).

**FIGURE 5:**
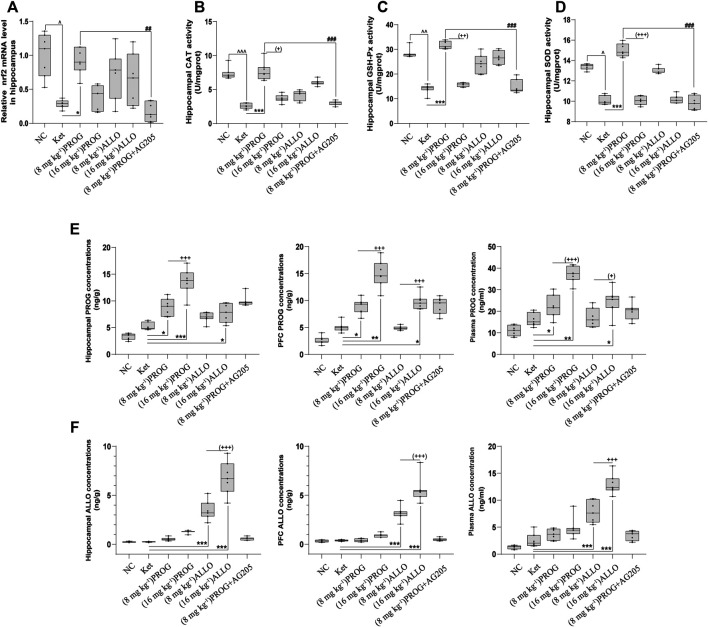
Hippocampal Nrf2 mRNA expression and the enzyme activity of CAT, SOD, GSH-Px and the concentrations of PROG and ALLO in hippocampus, PFC and plasma. **(A)** Nrf2, **(B)** CAT, **(C)** GSH-Px, **(D)** SOD, **(E)** PROG concentrations, **(F)** ALLO concentrations. ^*^ *p* < 0.05, ^**^ *p* < 0.01 and ^***^ *p* < 0.001 for add-on of PROG and ALLO vs. Ket. ^+^ *p* < 0.05, ^++^ *p* < 0.01 and ^+++^ *p* < 0.001 for PROG (16 mg kg^−1^) *vs* PROG (8 mg kg^−1^) and ALLO (16 mg kg^−1^) vs. ALLO (8 mg kg^−1^).

Furthermore, we examined the mRNA expression of Nrf2 and the activity of antioxidant enzymes in the hippocampus and PFC. The quantification of antioxidant enzymes acts as an indirect measure of oxidative stress. As illustrated in Figures 5A–D, compared with NC group, ketamine markedly reduced the mRNA expression of Nrf2 (*H* = 29.06, *p* < 0.0001; post hoc *p* = 0.0238), the activity of CAT (*H* = 42.91, *p* < 0.0001; post hoc *p* < 0.0001), GSH-Px (*H* = 41.75, *p* < 0.0001; post hoc *p* = 0.0060) and SOD (*H* = 38.71, *p* < 0.0001; post hoc *p* = 0.0425) in the hippocampus. Only PROG at 8 mg kg^−1^ ameliorated ketamine-induced decreases in Nrf2 mRNA expression (*p* = 0.0453) and CAT (*p* < 0.0001), GSH-Px (*p* < 0.0001), SOD (*p* = 0.0006) activity. Synchronous changes in the PGRMC1 signaling pathway also occurred in the PFC, and the results are shown in Supplementary Figure S3.

### Involvement of PGRMC1 in the Reversal Effects of PROG on Ketamine-Induced Cognitive Impairment

To confirm the regulation of PROG-mediated neuroprotection by PGRMC1, PROG-treated rats were co-administrated with AG205, which significantly weakened the protective effects of PROG against ketamine-induced cognitive impairment. On one hand, rats treated with the coadministration of AG205 and PROG (8 mg kg^−1^) exhibited poorer cognitive performance manifested as prolonged escape latencies (an effect of treatment [*F* (6, 420) = 343.2, *p* = 0.0002], an effect of time [*F* (2, 420) = 857.7, *p* < 0.0001] and an interaction between factors [*F* (12, 420) = 50.30, *p* < 0.0001] in the MWM test compared with those of the PROG monotherapy group (Figure 2). On the other hand, the addition of AG205 abolished the neuroprotective effect of 8 mg kg^−1^ PROG against the ketamine-induced downregulated expression of the PGRMC1 signaling pathway as well as the changes in downstream molecules [AG205+PROG (8 mg kg^−1^) *vs* PROG (8 mg kg^−1^), all *p* < 0.05, Figures 3–5].

### Concentrations of PROG and ALLO in the Plasma, Hippocampus and PFC after Different Treatments

To investigate the relationship between the doses and therapeutic effects of PROG and ALLO, we analyzed the concentrations of PROG and ALLO in these tissues as depicted in Figure 5. Compared with NC group, ketamine-treated rats did not exhibit higher concentrations of PROG and ALLO in hippocampus, PFC and plasma. When compared to ketamine group, add-on treatments of PROG at 8 and16 mg kg^−1^ significantly increased the levels of PROG without affecting the levels of ALLO in hippocampus [*H* = 41.34, *p* < 0.0001; post hoc *p* = 0.0310 (PROG at 8 mg kg^−1^), *p* = 0.0007 (PROG at 16 mg kg^−1^)], PFC [*H* = 43.18, *p* < 0.0001; post hoc *p* = 0.0482 (PROG at 8 mg kg^−1^), *p* = 0.0017 (PROG at 16 mg kg^−1^)] and plasma [*H* = 39.53, *p* < 0.0001; post hoc *p* = 0.0375 (PROG at 8 mg kg^−1^), *p* = 0.0023 (PROG at 16 mg kg^−1^)]. As expected, ALLO concentrations in these tissues were markedly elevated in both ALLO treated groups at 8 mg kg^−1^ (all *p* < 0.001) and 16 mg kg^−1^ (all *p* < 0.001) vs. ketamine group. Interestingly, ALLO treatment at 16 mg kg^−1^ also significantly increased the levels of PROG in hippocampus (*p* = 0.0100), PFC (*p* = 0.0113) and plasma (*p* = 0.0453) as compared with ketamine group.

## Discussion

Several key findings emerged in the present study. First, in basal conditions, both PROG and AG205 administration showed inhibitory effect on PGRMC1 expression in hippocampus and PFC as well as impaired MWM performance in AG205-treated rats. Second, ketamine significantly impaired hippocampal-dependent memory performance of the rats in the MWM test and downregulated the PGRMC1 pathway. Third, the cognitive impairment induced by ketamine was reversed by PROG and ALLO add-on treatments, and the PGRMC1/EGFR/GLP-1R/PI3K/Akt pathway was upregulated. Fourth, the coadministration of AG205 abolished the efficacy of PROG in the MWM test and offset the regulatory effect of PROG on the abovementioned PGRMC1 signaling pathway. Finally, high dose of ALLO administration led to increased concentrations of PROG in the plasma and brain, and this may be able to explain some of its neuroprotective efficacy.

### Effects of PROG, ALLO and AG205 on PGRMC1 Expression in Basal Conditions

In healthy status, overload of exogenous PROG/ALLO administration does appear to impair non-social learning and memory tasks in normal rodents (Johansson et al., 2002; Bychowski and Auger, 2012), which is in accordance with the previous observation that administration of PROG represses PGRMC1 transcription in rats *in vivo*. (Losel et al., 2008; Zhang et al., 2014). However, when ketamine-induced neurotoxicity and resulting suppression of PGRMC1 happen, add-on PROG may switch its role and exert the neuroprotective function via upregulation of PGRMC1 at an appropriate dose. So far, the therapeutic effect of RPOG has not been reported in ketamine-induced neurotoxicity, apart from the combination of estrogen and PROG in ketamine-induced disrupted PPI in rats (Van den Buuse et al., 2015). The discrepancy of PROG actions was possibly due to different basal conditions (healthy vs. injured) before PROG treatment, which warrants further exploration in future studies. More interest has been aroused to explore whether the neuroprotective effect of PROG can be observed when an injury or deficiency is present, as in the case of sub-chronic ketamine administration.

### Effects of PROG and ALLO on Cognitive Deficits Induced by Ketamine

Ketamine impairs cognition in both humans and animals (Moosavi et al., 2012; Parwani et al., 2005), which can model cognitive deficits associated with schizophrenia. It has been reported that cognitive impairment induced by sub-chronic ketamine exposure (30 mg kg^−1^ per day for five consecutive days) remains stable for 21 days (Rushforth et al., 2011). The MWM task was selected based on the extensive literatures (Zheng et al., 2015; Sun et al., 2016) indicating that NMDA receptor antagonists produce impairments in the MWM test.

Consistent with the emerging evidence, the data in our study showed that rats exposed to sub-chronic ketamine for five consecutive days exhibited impaired cognitive performance. It has been previously reported that PROG exerts neuroprotective effects in glutamate toxicity models (Van den Buuse et al., 2015) and improves spatial learning performance in the MWM test after traumatic brain injury (Djebaili et al., 2004; Jones et al., 2005). In the present experiment, we observed that PROG and ALLO were both able to attenuate ketamine-induced cognitive impairment in the MWM test.

### PROG and ALLO Reversed the Inhibitory Effects of Ketamine on the PI3K/Akt Pathway and Its Downstream Molecules

It has been reported that decreased activity of the PI3K/Akt pathway can, at least in part, explain the cognitive deficits in schizophrenia (Zheng et al., 2012; Zuo et al., 2016). Based on the fact that the level of Akt is significantly decreased in schizophrenia (Zheng et al., 2012), our present study also revealed the downregulation of the PI3K/Akt pathway in sub-chronic ketamine-exposed rats with cognitive impairment. Preclinical evidence has also revealed that the phosphorylation of Akt, which is crucial for PI3K-mediated memory enhancement via boosting cell survival and protein synthesis, can be elicited by PROG (Singh, 2001; Labombarda et al., 2003). Therefore, it is inferred that an increase in PI3K/Akt may be a contributing mechanism to improvements in spatial learning by PROG. Interestingly, our data showed that both PROG and its active metabolite ALLO increased the expression of the PI3K/Akt signaling pathway.

CREB, as a key transcriptional regulator, participates in multiple critical functions of the brain, including learning and cell survival (Peltier et al., 2007). Akt is capable of activating CREB, which then improves the expression of BDNF in pro-survival signaling (Aguiar et al., 2011). BDNF is thought to be a key regulator of learning and memory, which is involved in the pathogenesis of schizophrenia and is especially related to cognitive deficits (Nieto et al., 2013). Previous studies have suggested that ketamine impairs the learning and memory ability of rats and simultaneously markedly reduces the protein expression of p-Akt, p-CREB and BDNF (28 kDa, also called truncated BDNF) (Zuo et al., 2016). In addition, activated CREB and BDNF can further modulate the transcription process in the cell survival mechanism to ameliorate the neurotoxicity induced by ketamine (Zuo et al., 2016). In accordance with our study, cogent evidence has revealed that PROG (Singh and Su, 2013) and its active metabolite ALLO (Nin et al., 2011) can strengthen cognitive function by upregulating the expression of BDNF. Unlike truncated BDNF, another form of BDNF (14 kDa, called mature BDNF) was markedly downregulated by PROG and ALLO and upregulated by ketamine. Consistently, data (Carlino et al., 2011) has revealed an increase in the level of mature BDNF and a reduction in truncated BDNF in patients with schizophrenia with cognitive deficits. Evidence has suggested (Carlino et al., 2011) that truncated BDNF has similar properties as those of pro-BDNF (the precursor of BDNF) and can serve as an alternative of the inactive form of pro-BDNF, leading to an increase in pro-BDNF. Furthermore, cognitive impairment can mostly induce a compensatory increase in the processing of pro-BDNF to generate mature BDNF. This may provide an explanation for the reduction in truncated BDNF and the upregulation of mature BDNF in the ketamine group.

Caspase-3 and -9 belong to a family of cysteinyl-aspartate-specific proteases involved in apoptotic cell death. Previous studies have shown that prolonged ketamine exposure significantly increases cleaved caspase-3 and -9 levels, hence providing direct evidence for the activation of the intrinsic apoptotic pathway (Zou et al., 2009; Liu et al., 2013). In addition, another study (Djebaili et al., 2004) showed that, compared with vehicle alone, ALLO (16 mg kg^−1^) and PROG (8 mg kg^−1^) are able to decrease cleaved caspase-3 compared in injured rats.

Nrf2, a crucial regulator of oxidative stress, is activated by PI3K/Akt signaling (Lee et al., 2014). Reduced activity of Nrf2 and related antioxidant enzymes, including SOD, CAT and GSH-Px, have been observed in ketamine-exposed rats (Zugno et al., 2014). PROG reportedly protects neuronal cells from oxidative stress by upregulating antioxidative enzymes (Sharma et al., 2011).

Consistent with the evidence above, we confirmed that ketamine produced a significant increase in cleaved caspase-3 and caspase-9 and a decrease in CREB, BDNF and Nfr2, whereas coadministration with PROG or ALLO ameliorated these changes (Figure 3). Therefore, it is suggested that the therapeutic effects of PROG and ALLO are associated with their ability to influence CREB, BDNF, caspase-3/9 and Nrf2 expression to protect against ketamine-induced neurotoxicity.

### Add-On PROG and ALLO Treatments Reversed the Suppression of EGFR and GLP-1R Induced by Ketamine

EGFR and GLP-1R are both positive regulators of the PI3K/Akt pathway. Studies have shown that exendin-4 (an agonist of GLP-1R) inhibits neuronal apoptosis by upregulating the GLP-1R/PI3K/Akt signaling pathway (Xie et al., 2018). Preclinical evidence has also suggested that GLP-1R in the brain represents a promising new target for both cognitive-enhancing and neuroprotective agents (During et al., 2003). Similarly, it has also been reported that an adenosine A_1_ receptor agonist can activate the EGFR/PI3K/Akt pathway to mediate neuroprotection in cortical neurons (Xie et al., 2009). It is also known that EGFR can mediate Nrf2 signaling activation by several other stimuli in the process of neuroprotection (Gu et al., 2015). Although few studies have investigated the effect of ketamine on EGFR and GLP-1R, it has been demonstrated that neuroprotection mediated by EGFR and GLP-1R can be abolished by using selective kinase inhibitors (Xie et al., 2009). In support of the evidence above, our data also provides evidence that PROG and ALLO exert their neuroprotective effects against ketamine by upregulating EGFR and GLP-1R signaling and downstream molecules.

### Role of PGRMC1 in the PROG-Mediated Therapeutic Effects

**FIGURE 6:**
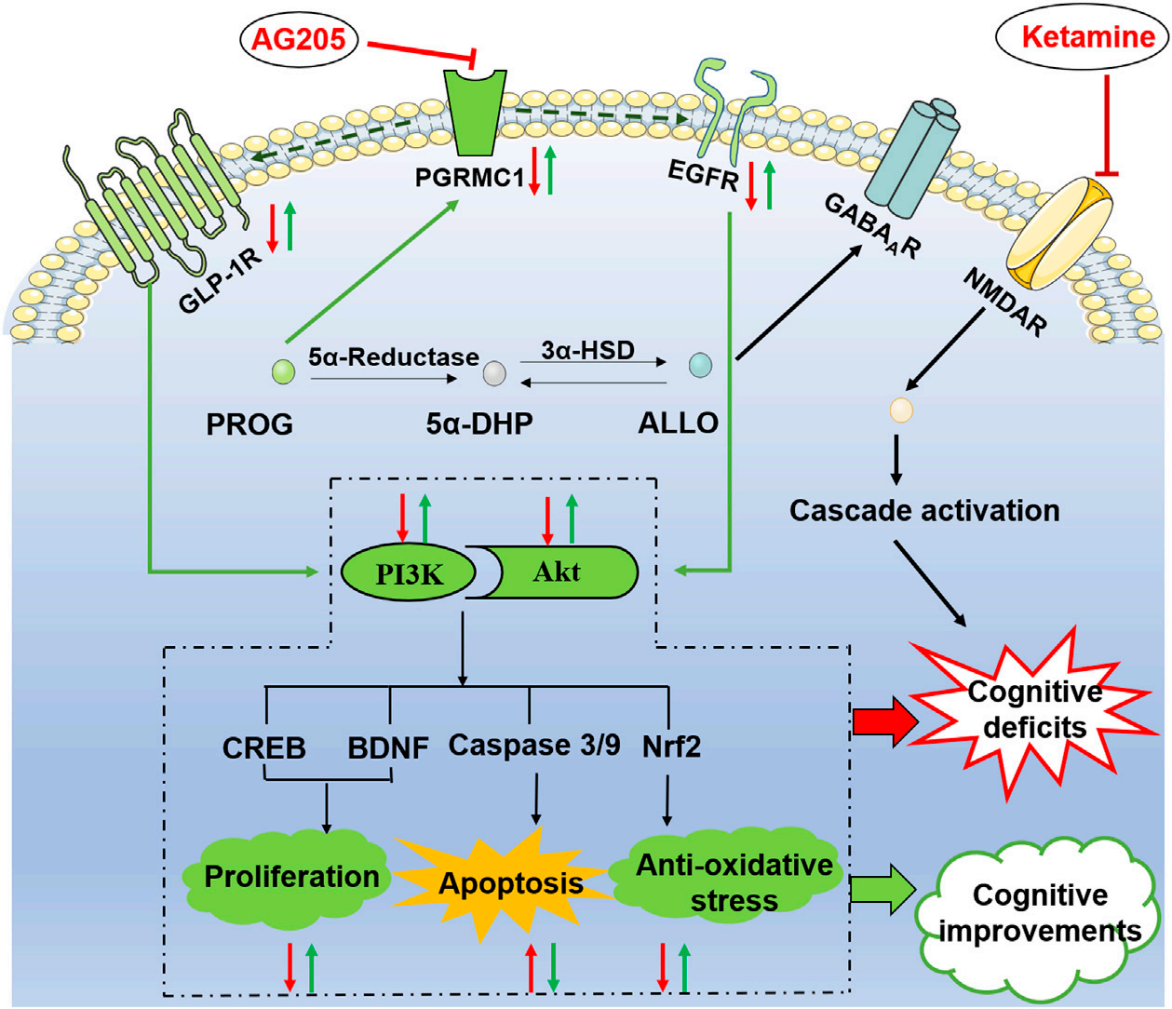
Illustrative model of the mechanism underlying the neuroprotective effects of PROG and ALLO against ketamine-induced cognitive deficits. Under certain conditions, the PI3K/Akt pathway can be activated to exert its neuroprotective function by phosphorylating a battery of protein substrates, including Nuclear factor erythroid-2-related factor 2 (Nrf2), caspase-3/9, cAMP response element-binding protein (CREB) and brain-derived neurotrophic factor (BDNF). Furthermore, the PI3K/Akt pathway is a putative downstream signaling pathway regulated by EGFR and GLP-1R to elicit multiple biological responses, especially cognitive function. Intriguingly, PGRMC1 co-precipitates and co-localizes with EGFR in cytoplasmic vesicles in cells and also serves as a novel component of the liganded GLP-1R complex. In the present study, the therapeutic effect of PROG or ALLO at least in part rely on the activation of PGRMC1/EGFR/GLP-1R/PI3K/Akt pathway in the brain. Moreover, the PRGMC1-specific inhibitor AG205 significantly antagonized the therapeutic effects of PROG in the MWM task and downregulated the PGRMC1/EGFR/GLP-1R pathway, which supports a role for PGRMC1 in the functional regulation of EGFR and GLP-1R, as well as their relation to neuroprotective effects.

As illustrated in Figure 6, PGRMC1 has the ability to interact with EGFR and GLP-1R (Zhang et al., 2014). In the present study, the PRGMC1-specific inhibitor AG205 significantly antagonized the therapeutic effects of PROG in the MWM task and downregulated the PGRMC1/EGFR/GLP-1R pathway, which supports a role for PGRMC1 in the functional regulation of EGFR and GLP-1R (Zhang et al., 2014).

Consistent with our results, previous findings have also revealed that PGRMC1 protein levels are upregulated by PROG in the brain after TBI (Meffre et al., 2005). Although classic Pgr has multiple physiological functions, there is a lack of evidence suggesting that the neuroprotection of PROG is mediated by classic Pgr. Interestingly, our data demonstrate that cotreatment with AG205, a specific inhibitor of PGRMC1, reversed the neuroprotective effect of PROG. Given that PROG possesses a high affinity for PGRMC1, the data suggest that the neuroprotective effects of PROG against ketamine’s neurotoxicity are mediated by PGRMC1. Studies on the potential role of PGRMC1 as a participant in the antiapoptotic process of PROG in rats have been performed (Qin et al., 2015). Additionally, data (Su et al., 2012) have revealed that PROG can elicit the release of BDNF in glia via a PGRMC1-mediated signaling mechanism. Taken together, the evidence above suggests that the upregulation of PGRMC1 expression after an increase in brain PROG levels is associated with its neuroprotective mechanism.

### Neuroprotective Role of PGRMC1 Depends on the Conversion between PROG and ALLO

On one hand, ALLO is biosynthesized from PROG in the brain through 5α-reductase- and 3α-hydroxysteroid dehydrogenase-mediated reactions, which are irreversible and rate-limited by the 5α-reductase step (Dong et al., 2001). Due to its lipophilicity, peripheral ALLO can readily cross into brain (Paul and Purdy, 1992). In ALLO-treated groups, the concentrations of PROG are both endogenous and at the same baseline level before add-on ALLO treatments. Exogenous-administration of ALLO can interfere the normal conversion process from PROG to ALLO. In our study, high dose of ALLO provided higher exogenous concentration of ALLO, which could exert a much stronger interfering effect in the conversion process than the ALLO (8 mg kg^−1^) group, finally resulting in significantly elevated PROG levels in the ALLO (16 mg kg^−1^) group. This fully explains the higher level of PROG observed in the ALLO (16 mg kg^−1^) group than in the ALLO (8 mg kg^−1^) group (Figure 5).

Consistently, ALLO (16 mg kg^−1^) also exhibited a stronger therapeutic effect in regulating the PGRMC1 pathway. On the other hand, ALLO does not bind to classical intracellular PROG receptors and exerts its neuroprotective actions via the modulation of membrane-associated GABA_A_ receptor sites (Ishihara et al., 2013). Additionally, a clinical finding (Cai et al., 2018b) suggested that low levels of ALLO may lead to weaker neuroprotection or that excessive levels of ALLO are involved in neuroprotection against excitotoxic damage in patients with schizophrenia. This evidence suggests the possibility of a dual action of ALLO in the present study, i.e. directly via GABA_A_ receptors at a lower ALLO and via attenuating the conversion process simultaneously at high level of ALLO resulting in an increased level of PROG in the brain to directly activate PGRMC1. Intriguingly, a low dose of PROG did not alter ALLO levels in these tissues and exhibited stronger therapeutic effects than a low dose of ALLO, which suggests that the neuroprotective effect of PROG is mostly mediated by PROG itself, not via its metabolite ALLO.

Reports (Goss et al., 2003) have indicated that low and moderate doses of PROG (8, 16 mg kg^−1^) are beneficial for facilitating behavioral recovery, while a high dose (32 mg kg^−1^) is either ineffective or potentially harmful in the MWM test in rats. In accordance with this finding, our data also support that either 8 or 16 mg kg^−1^ PROG possesses therapeutic effects. However, the fact that 8 mg kg^−1^ PROG displayed better therapeutic efficiency than 16 mg kg^−1^ PROG cannot be ignored and greatly aroused our interest. First, previous studies on PROG dose-response were conducted in a model of TBI, while our study mimicked the ketamine-induced schizophrenia; this difference probably led to complexities and differences in PROG dose-response. Second, the accumulation of PROG was reported to possibly mediate its suppressive effect on PGRMC1 expression (Losel et al., 2008), which may result in reduced neuroprotection and even further lead to neurotoxicity under some circumstances. In agreement with our hypothesis, previous studies have reported that subcutaneous injection of PROG at a lower dose acutely or sub-chronically impairs social recognition memory of normal rats both in a social discrimination task and in a spatial task (Sun et al., 2010; Bychowski and Auger, 2012). Nevertheless, the therapeutic differences in the doses of PROG remain unclear at this stage and require further elucidation.

### Limitation

Although prepulse inhibition (PPI) is used to analyze early attentional gating mechanisms in animal models of schizophrenia, the reasons why our study selected MWM test are as follows:

1. PPI deficits have been observed not only in schizophrenia but also in other neuropsychiatric disorders, in which an increased dopamine functioning is involved not in NMDA system (Geyer, 2006; Schellekens et al., 2010).
2. Animal models (Vargas et al., 2016) and clinical data (Dawson et al., 2000) both indicated that PPI deficits not only correlate with cognitive impairment such as working memory or alternation behavior, locomotion activity, but also some negative symptoms described in schizophrenia.

In view of lacking specificity, the PPI deficits is not suitable for evaluating the cognitive impairment modeled by ketamine. Nowadays, there is still controversy about the use of ketamine and other NMDAR inhibitors in well-establishment of schizophrenia models in animals. However, it is well-recognized that sub-chronic ketamine administration induced neurotoxicity in rodents manifested as cognitive impairments in the MWM test, as well as its relevance to schizophrenia-like cognitive deficits (Moosavi et al., 2012; Sabbagh et al., 2012). So far, a larger amount of preclinical researches (Sun et al., 2016; Lu et al., 2017; Li et al., 2019) have supported the application of MWM maze for the evaluation of cognitive deficits induced by ketamine. Take these factors into consideration, the MWM maze test is an appropriate choice for our experiment.

### Future Remarks

Evidence (Roof and Hall, 2000) suggests that the greater neuroprotection afforded to females is likely due to the effects of circulating estrogens and progestins. The neuroprotection provided by exogenous administration of these hormones extends to males as well. However, it is evident (Chen et al., 2018) that the efficacy of neurosteorids for cognitive function may differ between the genders. Moreover, neurosteroids may be involved in the gender differences found in the susceptibility to schizophrenia (Huang et al., 2017). In future clinical practice, it is recommended to measure the concentrations of PROG to optimize the therapeutic regimen based on gender differences.

## Conclusion

In conclusion, our study demonstrated that treatments with PROG or ALLO significantly ameliorate the cognitive impairment due to ketamine’s neurotoxicity. The therapeutic effects at least in part rely on the activation of PGRMC1/EGFR/GLP-1R/PI3K/Akt pathway in the brain. Co-administration with AG205, the specific inhibitor of PGRMC1, abolished the therapeutic effect of PROG on ketamine-induced neurotoxicity, verifying the key role of PGRMC1. Therefore, PROG or ALLO supplementation might be a potential therapeutic strategy to restore cognitive function in clinical practice. Specifically, the present study may shed light on future possibilities for neurosteroid treatments to enhance cognitive performance in neuropsychiatric diseases and for the development of pharmacological solutions to improve cognitive function by targeting on PGRMC1 signaling.

## Data Availability

The raw data supporting the conclusions of this article will be made available by the authors, without undue reservation.
