# Supplementary material for: A Potential Mechanism Underlying the Therapeutic Effects of Progesterone and Allopregnanolone on Ketamine-Induced Cognitive Deficits
Source: Front Pharmacol. 2021 Mar 8;12:612083. doi: 10.3389/fphar.2021.612083 (PMC7985688; doi:10.3389/fphar.2021.612083)
Supplement: Supplementary file 1 [file presentation1.pdf]

Supplementary Material

1 Supplementary Data

1.1 The algorithm used for calculation of AG205 dose

To compare the binding modes of AG205 and PROG on PGRMC1, a molecular docking analysis was performed. The crystal structure of human PGRMC1 (Protein Data Bank, PDB, http://www.rcsb.org/pdb, code: 4X8Y) was selected as the receptor for molecular analysis. The initial ligand molecule structures (AG205 and PROG) were retrieved from the NCBI-PubChem Compound Database. The docking simulation was performed with Schrodinger (Schrödinger, LLC, New York, NY, USA), a molecular modeling simulation program that is widely used for the automated docking of ligands and their macromolecular receptors. The conformation corresponding to the lowest energy was selected as the most probable binding conformation.

Molecular docking pattern and score of AG205 and PROG binding to the sits of SH2 and SH3 of PGRMC1 are obtained and shown in Supplementary Tables 1 and 2. Then, we selected 8 mg/kg PROG as a standard for AG205 dose conversion. To inhibit the binding effects of PROG to PGRMC1, the dose of the PGRMC1 specific inhibitor, AG205 was deduced by the molecular docking binding value as illustrated in Supplementary Table 3.

1.2 Supplementary Tables

Supplementary Table 1. Molecular docking pattern and score of ligand molecule binding to SH2 site of PGRMC1.

Ligand	Docking score	Interaction	Three-dimensional combination mode	Two-dimensional combination mode
AG205	-5.695	Pi-cat: N-Heterocycle ---Lys172<br>H-bond:<br>carbonyl group---Lys172	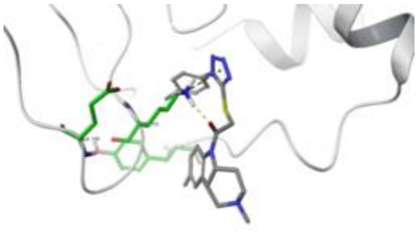	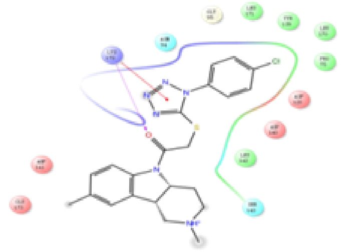
Progesterone	-3.433	none	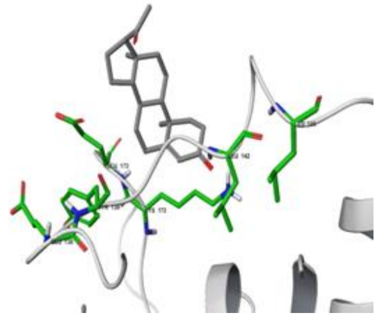	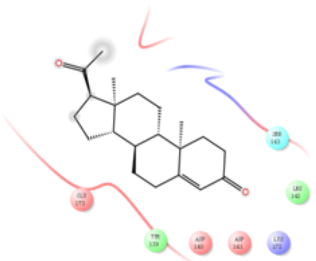

Supplementary Table 2. Molecular docking pattern and score of ligand molecule binding to SH3 site of PGRMC1.

Ligand	Docking score	Interaction	Three-dimensional combination mode	Two-dimensional combination mode
AG205	-5.794	Pi-cat: N-Heterocycle--Gly64	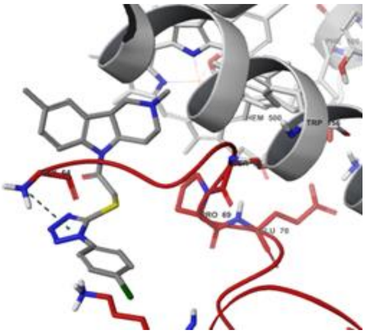	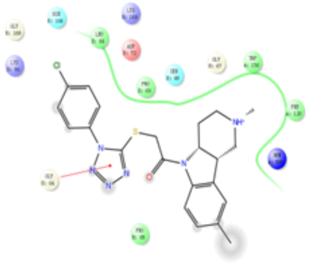
Progesterone	-3.814	none	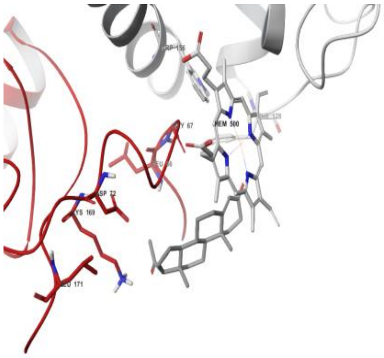	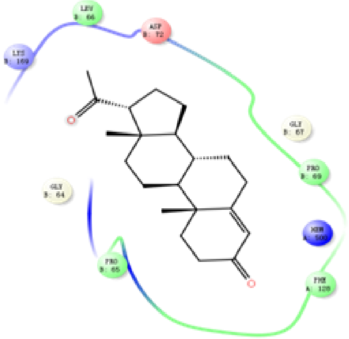

Supplementary Table 3. Parameters used for dose conversion.

	PROG	AG205
Molecular formula	$C_{21}H_{30}O_2$	$C_{22}H_{23}ClN_6OS$
Molecular weight	314.46	454.98
Docking score of SH_2 site (absolute value)	3.433	5.695
Docking score of SH_3 site (absolute value)	3.814	5.794
Total score	7.247	11.489
Molar ratio of PROG/AG205	1:1.585	
Dose	8 mg/kg	7.3 mg/kg

Note: The larger the docking score, the smaller the molar amount needed to achieve the same effect based on the docking theory.

2. Supplementary Figures

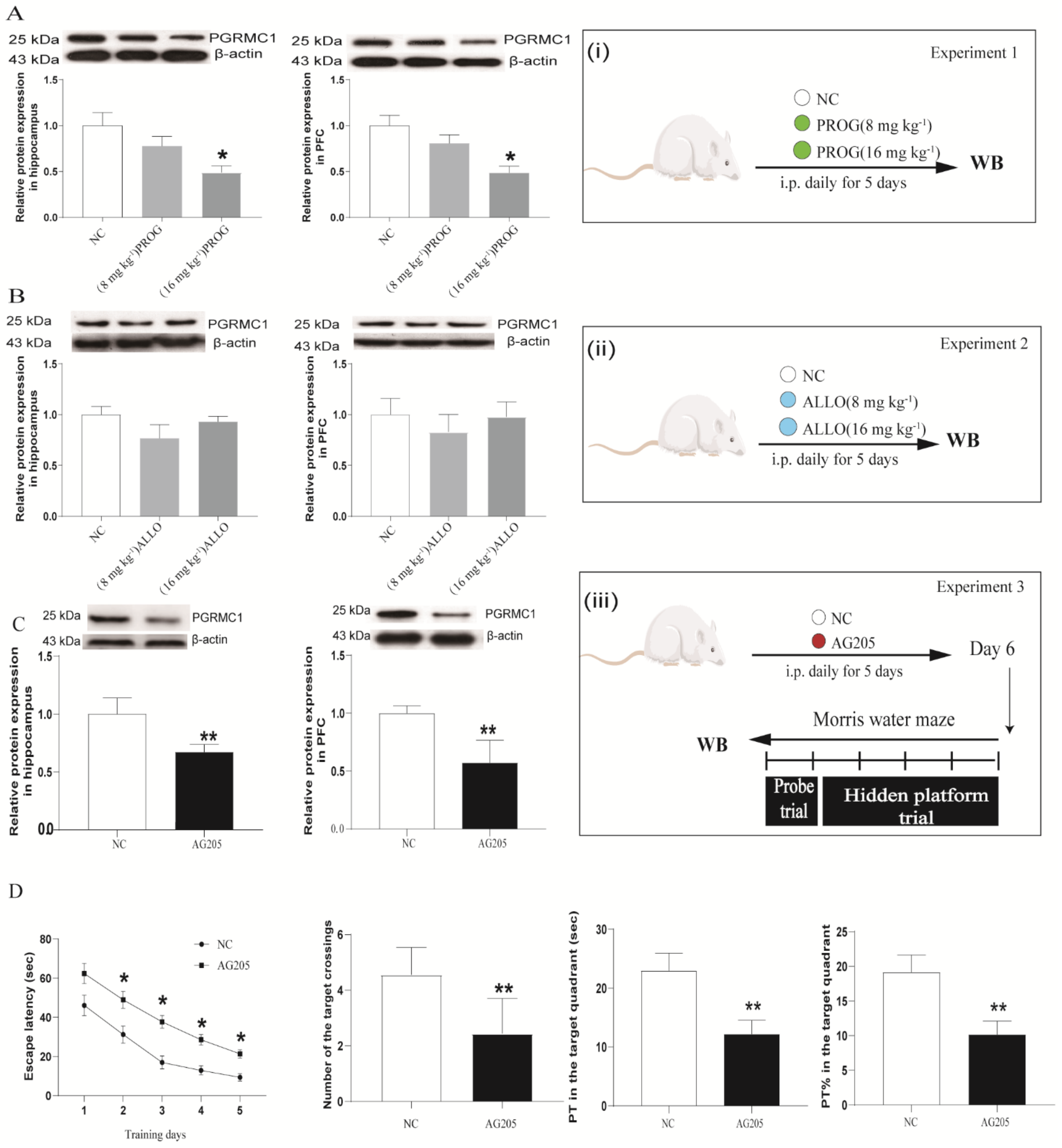

Supplementary Figure 1. Effects of PROG, ALLO and AG205 on PGRMC1 expression in basal conditions. The arrangements for experiment 1, 2 and 3 were illustrated in (**i**), (**ii**) and (**iii**). (**A**) Effects of PROG on PGRMC1 expression in hippocampus and PFC. (**B**) Effects of ALLO on PGRMC1 expression in hippocampus and PFC. Data are expressed as mean±SD, n=3 for each experimental group. *p<0.05 *vs* NC group. (**C**) Effect of AG205 on PGRMC1 expression in hippocampus and PFC. **(D)** Effect of AG205 on behavioral performance aspect including escape latency, the number of the target crossings, PT in the quadrant and the PT% in the quadrant. Data are expressed as mean±SD, n=6 for each experimental group. **p<0.01, *p<0.05 *vs* NC group.

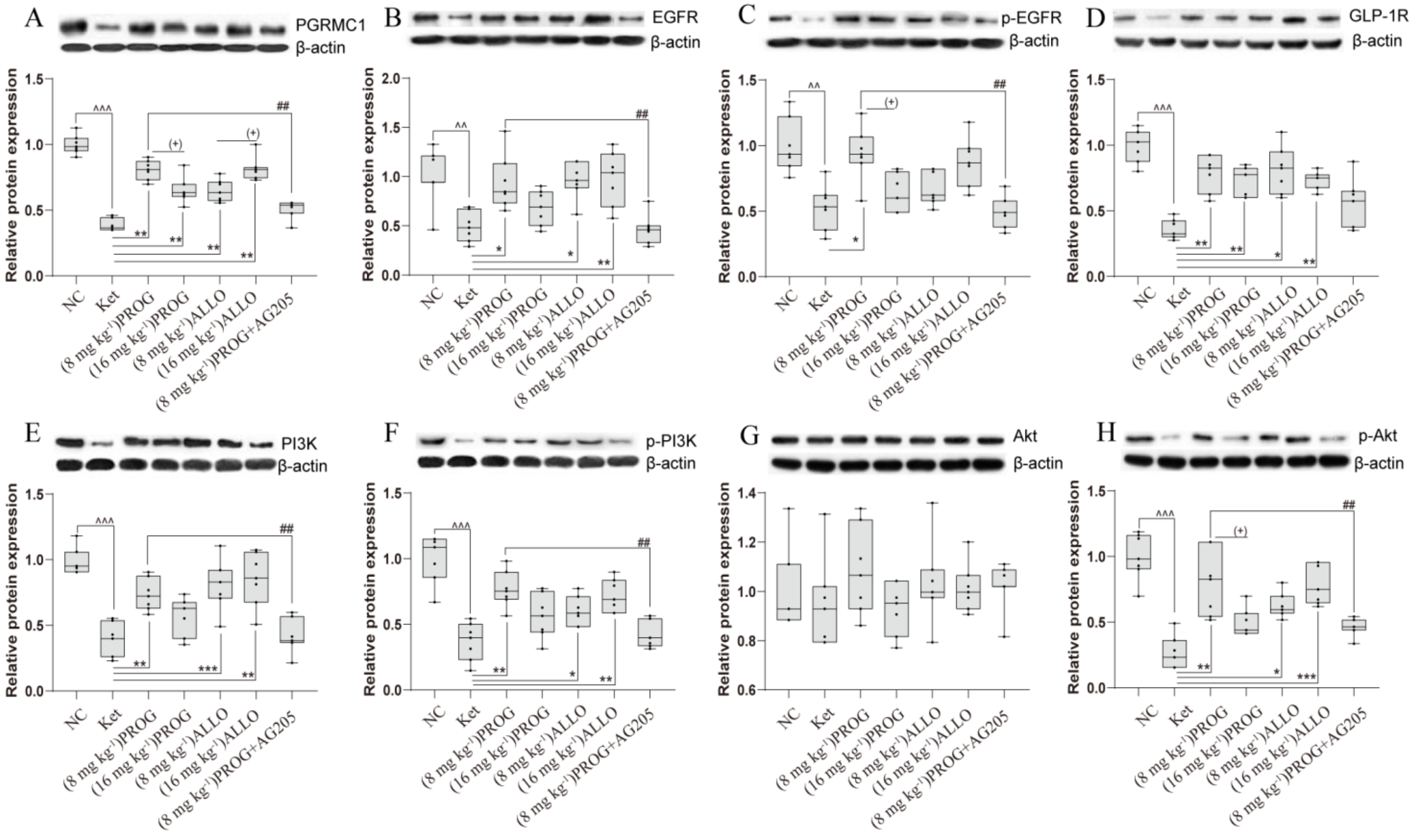

Supplementary Figure 2. Effects of ketamine and PROG, ALLO, AG205 add-on treatments on the PGRMC1/EGFR/GLP-1R/PI3K/Akt signaling pathway and related downstream signal molecules in the PFC. Western blot analysis of the protein expression: **(A)** PGRMC1 (H=42.01, p<0.0001), **(B)** EGFR (H=27.82, p=0.0001), **(C)** p-EGFR (H=30.74, p<0.0001), **(D)** GLP-1R (H=29.93, p<0.0001), **(E)** PI3K (H=33.59, p<0.0001), **(F)** p-PI3K (H=33.11, p<0.0001), **(G)** Akt (H=6.384, p=0.3816), **(H)** p-Akt (H=37.34, p<0.0001). Kruskal-Wallis one-way analysis of variance followed by post hoc Dunn's multiple comparisons test was used. $^{\wedge}p$<0.05, $^{\wedge\wedge}p$<0.01, and $^{\wedge\wedge\wedge}p$<0.001 for Ket *vs* NC. $^{*}p$<0.05, $^{**}p$<0.01 and $^{***}p$<0.001 for add-on of PROG and ALLO *vs* Ket. $^{\#}$p<0.05, $^{\#\#}$p<0.01 and $^{\#\#\#}p$<0.001 for PROG (8 mg kg^{-1})+AG205 *vs* PROG (8 mg kg^{-1}). ^{+}p<0.05, ^{++}p<0.01 and ^{+++}p<0.001 for PROG (16 mg kg^{-1}) *vs* PROG (8 mg kg^{-1}) and ALLO (16 mg kg^{-1}) *vs* ALLLO (8 mg kg^{-1}). Relative expression data were presented as the ratio to β-actin protein level. **Note:** In our study, the prototype protein and its phosphorylated form were separated in one gel simultaneously. Therefore, the loading control images of β-actin are re-used for illustrative purposes in B&C, E&F, G&H.

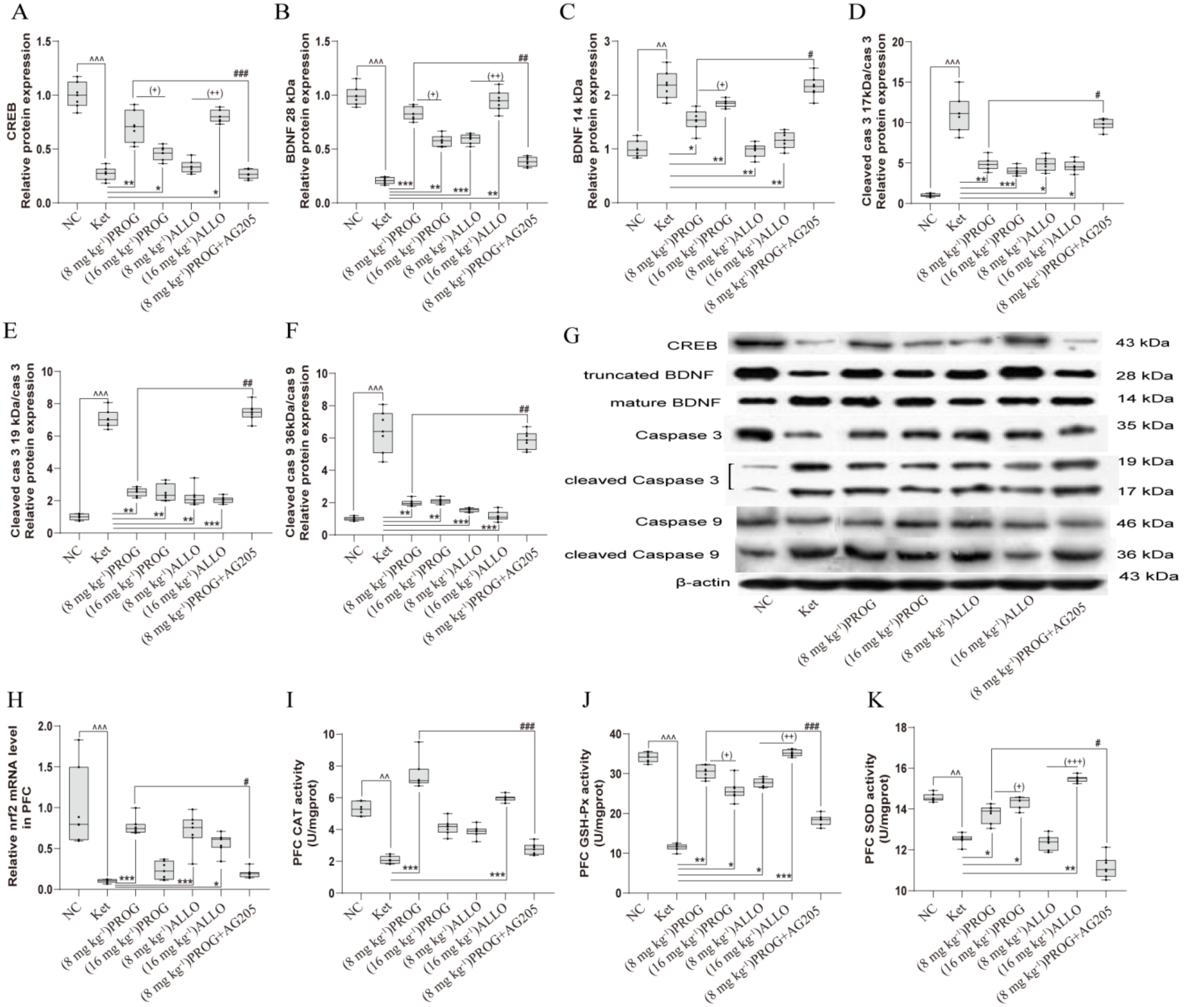

Supplementary Figure 3. Effects of ketamine and PROG, ALLO, AG205 add-on treatments on the related downstream signal molecules in the PFC. Western blot analysis of the protein expression: **(A)** CREB (H=43.27, p<0.0001), **(B)** truncated BDNF (H=44.71, p<0.0001), **(C)** mature BDNF (H=42.62, p<0.0001), **(D)** the ratio of cleaved caspase-3(17 kDa)/caspase-3 (H=39.79, p<0.0001), **(E)** the ratio of cleaved caspase-3 (19 kDa)/caspase 3 (H=40.73, p<0.0001), **(F)** the ratio of cleaved caspase-9/caspase-9 (H=44.19, p<0.0001). A representative blot is shown in **(G)**. Figure S3g-3k represent for relative Nrf2 mRNA levels and the activity of relevant anti-oxidative stress enzymes: **(H)** Nrf2 mRNA level (H=39.24, p<0.0001), **(I)** CAT activity (H=45.76, p<0.0001), **(J)** GSH-Px activity (H=45.22, p<0.0001), **(K)** SOD activity (H=44.84, p<0.0001). $^{\wedge}p$<0.05, $^{\wedge\wedge}p$<0.01, and $^{\wedge\wedge\wedge}p$<0.001 for Ket *vs* NC. $^{*}p$<0.05, $^{**}p$<0.01 and $^{***}p$<0.001 for add-on of PROG and ALLO *vs* Ket. $^{\#}p$<0.05, $^{\#\#}p$<0.01 and $^{\#\#\#}$p<0.001 for PROG (8 mg kg^{-1})+AG205 *vs* PROG(8 mg kg^{-1}). ^{+}p<0.05, ^{++}p<0.01 and $^{+++}$p<0.001 for PROG (16 mg kg^{-1}) *vs* PROG (8 mg kg^{-1}) and ALLO (16 mg kg^{-1}) *vs* ALLLO (8 mg kg^{-1}). Relative expression data were presented as the ratio to β-actin protein level.
